# Taxonomic study of the genus *Townesia* Ozols (Hymenoptera, Ichneumonidae, Pimplinae) with description of a new species from China and a key to world species

**DOI:** 10.3897/zookeys.878.38071

**Published:** 2019-10-07

**Authors:** Tao Li, Shu-Ping Sun, Mao-Ling Sheng, Jing-Xian Liu, Nhi Thi Pham

**Affiliations:** 1 General Station of Forest and Grassland Pest Management, National Forestry and Grassland Administration, No. 58 Huanghe North Street, Shenyang 110034, China National Forestry and Grassland Administration Shenyang China; 2 Department of Entomology, South China Agricultural University, Guangzhou 510642, China South China Agricultural University Guangzhou China; 3 Institute of Ecology and Biological Resources, Vietnam Academy of Science and Technology, 18 Hoang Quoc Viet, Hanoi, Vietnam Institute of Ecology and Biological Resources, Vietnam Academy of Science and Technology Hanoi Vietnam

**Keywords:** Ephialtini, host, host plant

## Abstract

Five species of the genus *Townesia* Ozols are reported. One, *Townesia sulcata* Sheng & Li, **sp. nov.** collected from Liaoning province, China, is new to science. In addition, digital images and a taxonomic key to the all species of *Townesia* are presented.

## Introduction

*Townesia* Ozols, 1962, belonging to the tribe Ephialtini of the subfamily Pimplinae (Hymenoptera, Ichneumonidae), comprises four species (Yu et al. 2016), of which two are from the Oriental Region (Gupta and Tikar 1976; Liu and He 2012), two from the Palaearctic Region and one from the Nearctic Region (Sheng and Sun 2010; Townes et al. 1960; Yu et al. 2016). Two species of *Townesia* were known from China (He et al. 1996; Sheng and Li 2006; Liu and He 2012). The diagnostic characters of the genus were most recently revised by Liu and He (2012) and Townes (1969).

Fourteen host species, belonging to Buprestidae and Cerambycidae (Coleoptera); Megachilidae, Siricidae, Sphecidae, and Tenthredinidae (Hymenoptera); and Sesiidae and Tortricidae (Lepidoptera) have been recorded (He et al. 1996; Sheng and Li 2006; Yu et al. 2016).

The aim of this article is to describe a new species and provide a key to the world species.

## Materials and methods

Specimens were collected with interception traps (IT) (Li et al. 2012) in Chagou, Haicheng, Liaoning Province, P.R. China. The type locality is a forest comprised of mixed deciduous angiosperms and evergreen conifers, mainly including *Quercus wutaishanica* Mayr, *Quercus* sp., *Larix* sp., *Castanea* spp., and *Pinus tabulaeformis* Carr.

Type specimens are deposited in the Insect Museum, General Station of Forest and Grassland Pest Management (**GSFGPM**), National Forestry and Grassland Administration, China.

The specimens of *Townesia tenuiventris* (Holmgren, 1860), deposited in the Zoologische Staatssammlung München, Germany, Germany (**ZSM**) and identified by Bauer, were examined and compared to the new species. The male characters of *T. tenuiventris* mentioned in the following key is based on Zwakhals’s (2010) description. The photos of holotype of *Townesia exilis* Gupta & Tikar, 1976, taken by Dr Jonathan Bremer (Florida Department of Agriculture and Consumer Services) was also checked.

Images of the new species were taken using a Stereomicroscope (Leica M205A) with a LAS Montage MultiFocus. Morphological terminology follows Broad et al. (2018).

## Taxonomy

### Townesia

Taxon classificationAnimaliaHymenopteraIchneumonidae

Ozols, 1962

825B2B2D-9B22-5A70-9CBE-C7C1430140D3

Townesia Ozols 1962: 12. Type species: Ephialtes tenuiventris Holmgren.

#### Diagnosis.

Teeth of mandible equal or almost equal. Epomia evidently present. Propodeum elongate, basal portion with median longitudinal impression; pleural carina complete. Areolet subtriangular, receiving *2m-cu* slightly distal of middle. Tarsal claws of female with a large basal lobe. Tergite I 0.6–0.7 times as long as tergite II (*T. exilis* same length), with lateral carina beneath spiracle; basal part of median dorsal carinae distinct. Tergites II to V elongate, with dense punctures. Ovipositor sheath longer than body length, usually about 2.5 times as long as forewing. Apical portion of ventral valve with distinct ridges, subapical portion without or with weak dorsal lobe (Figs 10–14).

### Key to world species of *Townesia*

**Table:** 

1	Female	**2**
–	Male	**6**
2	Tergite I as long as tergite II. Lateral tubercles of tergites III–V indistinct. Submetapleural carina complete. (Male unknown)	***T. exilis* Gupta & Tikar**
–	Tergite I distinctly shorter than tergite II. Tergites III–V with distinct lateral tubercles (except *T. cheni*). Submetapleural carina incomplete, or complete	**3**
3	Basal 0.6–0.7 of propodeum (Fig. 7) with median longitudinal furrow. Submetapleural carina complete. Forewing vein 1*cu-a* opposite *1/M*. Basal part of hind tibia (Fig. 1) light brown, subbasal part brownish black, median part unevenly yellowish brown, apically black. Basal parts of hind tarsomeres more or less buff. (Male unknown)	***T. sulcata* Sheng & Li, sp. nov.**
–	Propodeum without median longitudinal furrow, or with weak short furrow, not reaching middle of propodeum. Submetapleural carina only present anteriorly; if complete, then metasoma compressed. Forewing vein 1*cu-a* basal or distal of *1/M*. Hind tibia almost with same color, or basal portion slightly buff. Hind tarsomeres with same color	**4**
4	Forewing vein 1cu-a distal of *1/M*. Submetapleural carina complete. Third and subsequent tergites compressed. Apical portion of dorsal valve of ovipositor with fine teeth (Fig. 13). Hind coxae brownish black to black	***T. qinghaiensis* He**
–	Forewing vein 1cu-a basal of *1/M*. Submetapleural carina only present anteriorly. Metasoma uncompressed. Dorsal valve of ovipositor smooth (Fig. 11) or almost smooth, subbasal portion with two weak small tubercles, without distinct teeth (Figs 10, 14). Hind coxae reddish brown to dark brown	**5**
5	Forewing vein radius at middle of pterostigma. Tergites without lateral tubercles. Apical smooth transverse bands of tergites II and III approximately 0.15 times as long as respective length. Fore coxa yellow. Hind coxa dark brown. (Male unknown)	***T. cheni* Liu & He**
–	Forewing vein radius distinctly basal to middle of pterostigma. Tergites with distinct lateral tubercles. Apical smooth transverse bands of tergites II and III approximately 0.25 times as long as respective length. All coxae reddish brown	***T. tenuiventris* (Holmgren)**
6	Tergite I 2.3–2.5 times as long as apical width. Hind third tarsomere 1.1 times as long as first flagellomere. Hind fifth tarsomere 1.2–1.3 times as long as third tarsomere. Hind coxa black to brownish black	***T. qinghaiensis* He**
–	Tergite I 2.6–2.8 times as long as apical width. Hind third tarsomere 0.9 times as long as first flagellomere. Hind fifth tarsomere 1.0–1.1 times as long as third tarsomere. Hind coxa red	***T. tenuiventris* (Holmgren)**

### The species of *Townesia*

#### Townesia sulcata

Taxon classificationAnimaliaHymenopteraIchneumonidae

Sheng & Li sp. nov.

01FE591C-3251-55D2-8D38-8686B315AE7B

http://zoobank.org/B0DBB732-0E3D-4ECC-B9E6-148DD89D21E7

Figures 1–5 , 6–9 , 10

##### Diagnosis.

Face (Fig. 2) 1.2 times as wide as long. Malar space approximately 0.3 times as long as basal width of mandible. Postocellar line approximately 0.8 times as long as ocular-ocellar line. Antenna with 24 flagellomeres. Forewing vein *1cu-a* opposite to *1/M*. Areolet receiving *2m-cu* approximately at its posterior 0.2. Hind wing vein *1-cu* about as long as *cu-a*. Basal 0.6–0.7 of propodeum with median longitudinal furrow. Apical portion of ovipositor evenly downcurved.

##### Description.

**Figures 1–5.**
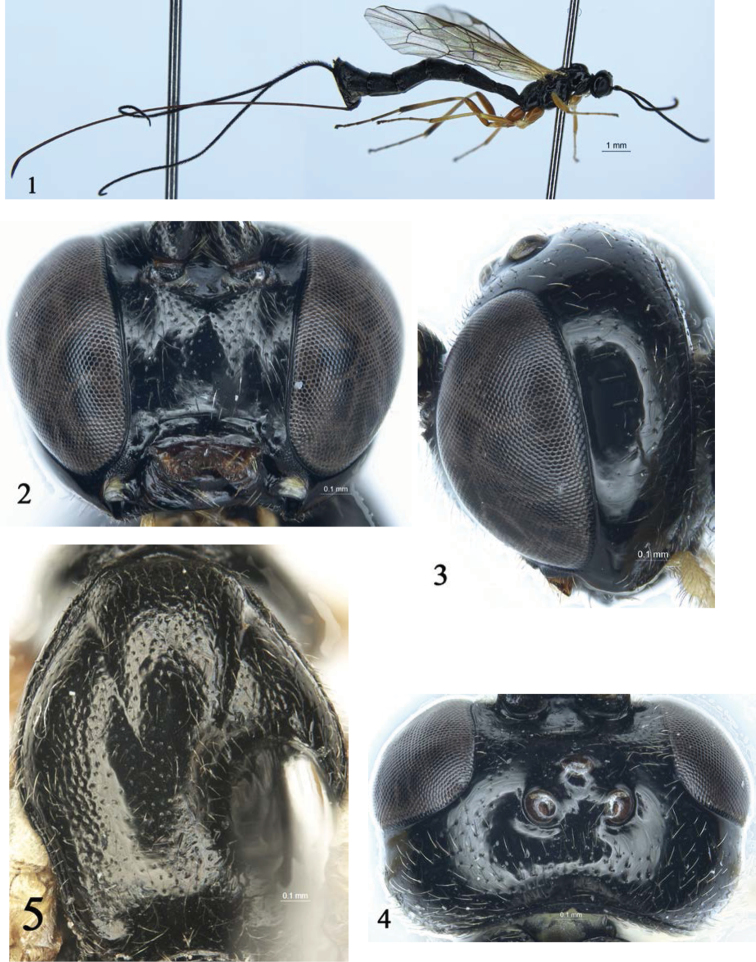
*Townesia sulcata* Sheng & Li, sp.nov. Holotype, ♀ **1** habitus, lateral view **2** head, anterior view **3** head, lateral view **4** head, dorsal view **5** mesoscutum.

Female (Fig. 1). Body length approximately 10.4–12.2 mm. Forewing length 7.1–7.8 mm. Ovipositor sheath 11.5–13.1 mm.

***Head.*** Face (Fig. 2) approximately 1.2 times as wide as long, smooth with sparse, fine punctures and white hairs; median portion slightly convex. Basal portion of clypeus smooth, with sparse, fine punctures and yellowish-brown hairs; apical portion with dense, fine punctures and short, yellowish-brown hairs; apical margin with deep, median concavity. Malar area with finely granulose texture. Malar space approximately 0.3 times as long as basal width of mandible. Inner orbits of compound eyes convergent downwards. Gena smooth (Fig. 3), with fine, sparse punctures and short, yellowish-brown hairs. Vertex (Fig. 4) smooth, laterally with very sparse punctures, hind part with relatively dense punctures and white hairs. Postocellar line approximately 0.8 times as long as ocular-ocellar line. Upper part of frons with evenly dense punctures and short, white hairs; lower portion concave, with weak, fine, transverse wrinkles. Antenna with 24 flagellomeres, ratio of length from first to fifth flagellomeres: 1.2:1.0:1.0:0.9:0.8. Occipital carina complete.

**Figures 6–9.**
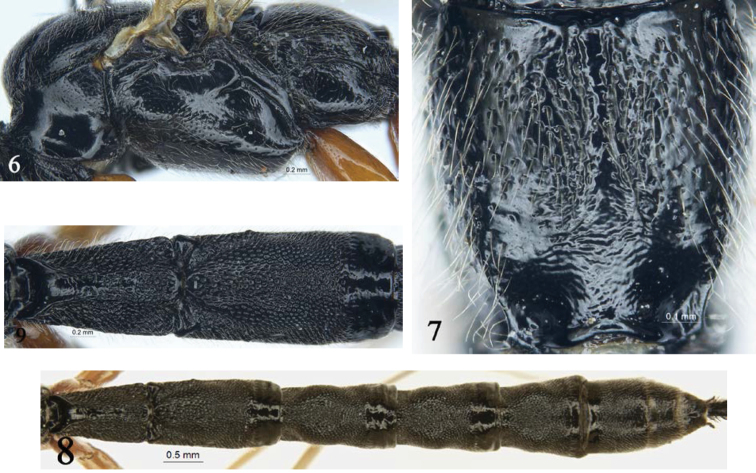
*Townesia sulcata* Sheng & Li, sp.nov. Holotype, ♀ **6** mesosoma, lateral view **7** propodeum **8** metasoma, dorsal view **9** tergites I–II, dorsal view.

***Mesosoma.*** Pronotum (Fig. 6) smooth; upper and upper-posterior portions with fine punctures and short, white hairs. Mesoscutum (Fig. 5) slightly convex, with dense punctures and yellowish-white hairs, distance between punctures 0.5–3.5 times diameter of puncture, posterior portion distinctly more sparsely punctate than anterior portion. Notaulus evident, reaching about 0.6 the distance to posterior margin of mesoscutum. Scutellum slightly convex, with dense punctures. Posterior portion of postscutellum transversely convex, with sparse punctures; anterior portion oblique concavity. Mesopleuron (Fig. 6) smooth; anterior and lower portions with dense punctures and grey-white hairs. Speculum with few hairs. Metapleuron with sparse punctures. Submetalpeural carina complete. Wings yellowish hyaline; fore wing with vein *1cu-a* opposite to *1/M*. Areolet oblique quadrangle, receiving *2m-cu* approximately at its posterior 0.2. Hind wing vein *1-cu* about as long as *cu-a*. Claws with large basal lobe. Ratio of length of hind first to fifth tarsomeres: 9.5:5.0:3.1:1.0:2.5. Propodeum (Fig. 7) smooth, shiny, with uneven punctures and yellowish-white hairs; apical median portion smooth; basal 0.6–0.7 with median longitudinal furrow; spiracle small, circular.

**Figures 10–14.**
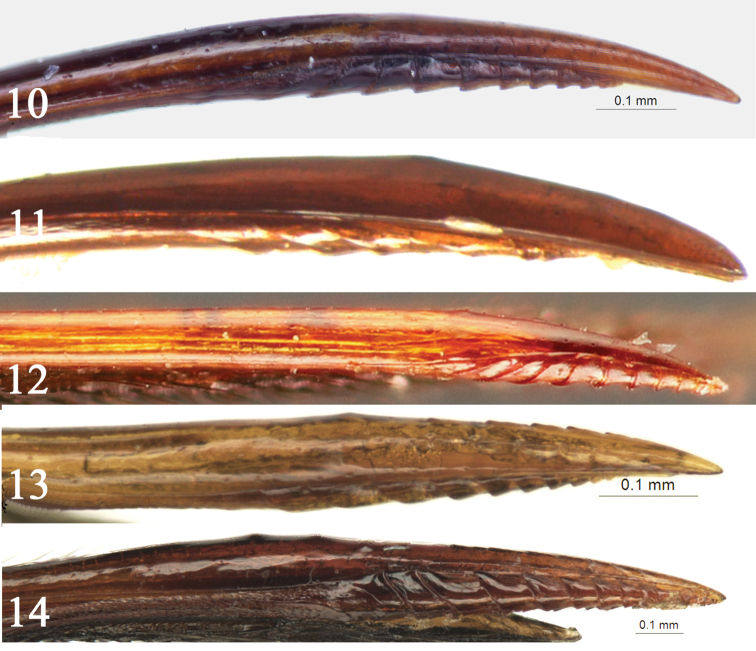
Apical part of ovipositor, lateral view **10***Townesia sulcata* Sheng & Li, sp. nov. **11***Townesia cheni* Liu & He **12***Townesia exilis* Gupta & Tikar **13***Townesia qinghaiensis* He **14***Townesia tenuiventris* (Holmgren).

***Metasoma*** (Fig. 8). Tergite I (Fig. 9) about 1.7 times as long as apical width; with dense punctures; median portion obviously raised, median longitudinal portion weakly concave; spiracle small, circular, located as basal 0.4 of tergite I. Tergite II (Fig. 9) about 1.9 times as long as apical width; with same texture as that of tergite I, apical 0.25 glabrous. Tergites III–V with the same texture as that of tergite II, subbasal weakly concave, with lateral tubercles, apical 0.25 glabrous. Tergites VI–VII with dense punctures. Ovipositor slender, apical portion decurved, dorsal valve smooth, ventral valve with 10 or 11 ridges (Fig. 10).

***Coloration*** (Fig. 1). Black, except following. Maxillary and labial palpi, tegula, and basal portion of fore wing, yellow. Clypeus fusco-testaceous. Legs reddish brown. Fore and mid trochanters yellow; tibia yellowish brown; first to fifth tarsi brown; fifth tarsus dark brown. Hind trochantellus yellow; main portion of tibia yellowish brown to brown, apical portion dark brown; tarsus dark brown to black brown. Veins and pterostigma brown.

##### Male.

Unknown.

##### Etymology.

The name of the new species is based on the form of the propodeum which is characterized by having a median, longitudinal groove.

##### Type material.

Holotype: ♀, CHINA, Hongqiling, Chagou, Haicheng City, Liaoning Province, 15 May 2015, leg. Mao-Ling Sheng. Paratypes: 4 ♀♀, same data as holotype except leg. Mao-Ling Sheng, Tao Li.

##### Host.

Unknown.

##### Distribution.

China.

##### Comments.

This new species is similar to *T. qinghaiensis* He, 1996 in having the gena, vertex, mesopleuron (Fig. 6), and propodeum smooth and shiny; propodeum almost without transverse wrinkles; and main portion of hind tibia yellowish brown to brown, with subbasal and apical portion dark brown to black. It can be distinguished from the latter by the following combination of characters: forewing vein 1cu-a opposite *1/M* (distal in *T. qinghaiensis*), basal 0.6 to 0.7 of propodeum with median longitudinal furrow (absent in *T. qinghaiensis*), apical portion of ovipositor distinctly curved, dorsal valve smooth, without ridge (Fig. 10), straight and dorsal valve with weak ridges in *T. qinghaiensis* (Fig. 13), hind coxa red-brown (brownish black in *T. qinghaiensis*). The new species can be distinguished from all other species by the key provided above.

#### Townesia cheni

Taxon classificationAnimaliaHymenopteraIchneumonidae

Liu & He, 2012

E54F7EA2-296E-5B8E-B649-1439074C1FB8

Figure 11

##### Diagnosis.

Forewing length 9.1 mm. Ovipositor sheath length 18.0 mm. Upper tooth of mandible as long as lower tooth. Propodeum with dense punctures and short hairs, without median longitudinal furrow. Tergite I 2.1 times as long as apical width, 0.77 times as long as tergite II. Tergites II–V almost without lateral tubercles. Tergite II as long as tergite III, apical smooth transverse bands 0.15 times as long as respective length. Apical portion of ovipositor evenly curved. Fore coxa yellow. Hind coxa dark brown.

##### Male.

Unknown.

##### Host.

Unknown.

##### Material examined.

1 ♀ (Holotype), CHINA: Zhejiang Province, Mt West Tianmu, Laodian-xianrending, 1150–1106 m, 17 May 1988, Xue-Xin Chen leg., coll. no. 882277.

#### Townesia exilis

Taxon classificationAnimaliaHymenopteraIchneumonidae

Gupta & Tikar, 1976

1A332D55-CFAF-5ADA-98C3-A050A45715F4

Figure 12

##### Diagnosis.

Female: Forewing length 8 mm. Ovipositor sheath length 15 mm. Face smooth and shiny. Malar space 0.2 times as long as basal width of mandible. Pronotum smooth, upper-posterior corner with sparse punctures. Fore wing with vein 1*cu-a* opposite to *1/M*. Areolet receiving *2m-cu* approximately at its posterior 0.1. Hind wing vein *1-cu* 0.67 as long as *cu-a*. Tergite I as long as tergite II. Tergite II 2.6 times as long as its maximum width, with distinct, dense, fine punctures; apical, smooth, transverse bands 0.25 times as long as length. Tergite III 2.5 times as long as its maximum width. Lateral tubercles of tergites III–V indistinct. Submetapleural carina complete. Tegula, fore, and middle legs yellow to yellow brown; hind coxa, trochanter, and femur red.

**Male.** Unknown.

##### Host.

Unknown.

##### Material examined.

1 ♀ (Holotype), INDIA: Kashmir, Gulmarg, 2600 m, 23 June 1966, J.K. Jonathan leg., coll. no. J166.

##### Distribution.

India.

#### Townesia qinghaiensis

Taxon classificationAnimaliaHymenopteraIchneumonidae

He, 1996

E4425E63-717A-5F31-9D5B-563BB1620DFF

Figure 13

##### Diagnosis.

Female: Forewing length 6.7–11.5 mm. Ovipositor sheath length 10.0–17.0 mm. Face, gena, and vertex smooth, shiny, with very sparse, fine punctures. Malar space 0.2 times as long as basal width of mandible. Antenna with 22–23 flagellomeres. Propodeum shiny, almost without carina, without median longitudinal furrow. Tergite I 1.7 times as long as apical width, 0.7 times as long as tergite II. Tergite II 1.2 times as long as tergite III, apical smooth transverse bands of tergites II–IV 0.18 times as long as respective length. Apical portion of dorsal valve of ovipositor (Fig. 13) with fine teeth. Maxillary and labial palpi gray-black.

Male: Forewing length 5.5–12.5 mm. Antenna with 22–24 flagellomeres. Fore wing with vein 1*cu-a* slightly distal of or opposite to *1/M*. Hind third tarsomere 0.4 times as long as first tarsomere, 0.75–0.8 times as long as fifth tarsomere. Tergite I 2.3–2.5 times as long as apical width, 0.67–0.7 times as long as tergite II. Hind coxa black to brownish black.

##### Host.

*Cydia strobilella* (Linnaeus, 1758) (Lepidoptera, Tortricidae).

##### Host plant.

*Picea crassifolia* Kom., *P. mongolica* W.D. Xu.

##### Material examined.

4 ♀♀, 7 ♂♂, CHINA: Qilian, Qinghai Province, 16–26 April 2004, Mao-Ling Sheng. 68 ♀♀, 26 ♂♂, CHINA: Keshiketeng, Inner Mongolia, 6–10 April 2005, Mao-Ling Sheng. 1 ♂, CHINA: Aletai, Xinjiang, 30 March 2007, Mao-Ling Sheng. 1 ♀, CHINA: Mentougou, Beijing, 13 June 2008, Tao Wang.

##### Distribution.

China.

#### Townesia tenuiventris

Taxon classificationAnimaliaHymenopteraIchneumonidae

(Holmgren, 1860)

CA69DAA0-5505-57CB-A09C-EF4D9628474D

Figure 14

##### Diagnosis.

Female: Vertex smooth, shiny, lateral part with sparse, fine punctures. Postocellar line approximately 0.8 times as long as ocular-ocellar line. Upper-posterior part of mesopleuron smooth, shiny, without punctures. Forewing vein radius distinctly basal of middle of pterostigma; 1*cu-a* distinctly basal of *1/M*. Areolet receiving *2m-cu* approximately at its posterior 0.3. Tergite II 1.9 times as long as its maximum width, apical smooth transverse bands of tergites II 0.25–2.7 times as long as length. Subapical portion of upper valve of ovipositor with two indistinct tubercles; ventral valve with 10 or 11 ridges, basal 4 ridges strongly inclivous. All coxae, trochanters, and femora reddish brown.

##### Host.

Fourteen host species were recorded (Yu et al. 2016).

##### Material examined.

The specimens, deposited in ZSM and identified by Bauer, were examined.

##### Distribution.

Austria, Belarus, Belgium, Bulgaria, Canada, Denmark, Finland, France, Germany, Hungary, Ireland, Italy, Japan, Latvia, Lithuania, Netherlands, Norway, Poland, Romania, Russia, Serbia, Spain, Sweden, U.S.A., United Kingdom, Yugoslavia.

## Supplementary Material

XML Treatment for Townesia

XML Treatment for Townesia sulcata

XML Treatment for Townesia cheni

XML Treatment for Townesia exilis

XML Treatment for Townesia qinghaiensis

XML Treatment for Townesia tenuiventris
